# Supplementary material for: Machine learning-driven multifunctional peptide engineering for sustained ocular drug delivery
Source: Nat Commun. 2023 May 2;14:2509. doi: 10.1038/s41467-023-38056-w (PMC10154330; doi:10.1038/s41467-023-38056-w)
Supplement: Supplementary file 1 — Supplementary Information [file 41467_2023_38056_MOESM1_ESM.pdf]

Machine learning-driven multifunctional peptide engineering for sustained ocular drug delivery

Supplementary Information

Henry T. Hsueh[1,2*], Renee Ti Chou[3*], Usha Rai[1,4], Wathsala Liyanage[1,4], Yoo Chun Kim[1,4], Matthew B. Appell[1,5], Jahnavi Pejavar[1,2], Kirby T. Leo[1,6], Charlotte Davison[1,2], Patricia Kolodziejski[1,2], Ann Mozzer[1,4], HyeYoung Kwon[1,2], Maanasa Sista[1,7], Nicole M. Anders[8], Avelina Hemingway[8], Vishnu Rompicharla[1,4], Malia Edwards[4], Ian Pitha[1,4], Justin Hanes[1,2,4–6,8], Michael P. Cummings[3†], and Laura M. Ensign[1,2,4–6,8†]

[1]Center for Nanomedicine at the Wilmer Eye Institute, Johns Hopkins University School of Medicine, Baltimore, MD, USA
[2]Department of Chemical & Biomolecular Engineering, Johns Hopkins University, Baltimore, MD, USA
[3]Center for Bioinformatics and Computational Biology, University of Maryland, College Park, College Park, MD, USA
[4]Department of Ophthalmology, Johns Hopkins University School of Medicine, Baltimore, MD, USA
[5]Department of Pharmacology and Molecular Sciences, Johns Hopkins University, Baltimore, MD, USA
[6]Department of Biomedical Engineering, Johns Hopkins University, Baltimore, MD, USA
[7]Department of Biomedical Engineering, Case Western Reserve University, Cleveland, OH, USA
[8]The Sidney Kimmel Comprehensive Cancer Center at Johns Hopkins University, Baltimore, MD, USA
[*]Equally contributing authors
[†]Corresponding authors. e-mail: lensign@jhmi.edu; mcummin1@umd.edu

Contents

1 Supplementary Notes **4**
1.1 Machine learning input data sets 4
1.2 Machine learning cross-validation results 4
1.3 Adversarial control machine learning cross-validation results 5

2 Supplementary Figures **7**

3 Supplementary Tables **20**

List of Figures

Supplementary Fig. 1 7
Supplementary Fig. 2 8
Supplementary Fig. 3 9
Supplementary Fig. 4 10
Supplementary Fig. 5 11
Supplementary Fig. 6 12
Supplementary Fig. 7 13
Supplementary Fig. 8 14
Supplementary Fig. 9 15
Supplementary Fig. 10 16
Supplementary Fig. 11 17
Supplementary Fig. 12 18
Supplementary Fig. 13 19

List of Tables

Supplementary Table 1 20
Supplementary Table 2 20
Supplementary Table 3 20
Supplementary Table 4 21
Supplementary Table 5 22
Supplementary Table 6 23
Supplementary Table 7 24

1 Supplementary Notes

Supplementary materials, including the research notebook containing the code of the machine learning algorithms, have been deposited as a compressed folder (∼2.74 GB in total) in the Digital Repository at the University of Maryland (DRUM) with the identifier https://doi.org/10.13016/0jck-hnnv. To open the research notebook, download and decompress the folder, go to the subfolder `main_notebook`, and click on `index.html` to open the HTML document in a web browser, or click on `main_notebook.pdf` to open the PDF document. Data generated in this research have been stored in the subfolders `data` and `other_data`. The following shows the descriptions of the data files and their locations.

1.1 Machine learning input data sets

The `peptide_variable_descriptions.csv` file contains descriptions of peptide variables calculated in the input data sets for machine learning. The other data files are the machine learning input data sets, including pilot and second melanin binding peptide microarray data for training classification and regression machine learning models, as well as cell-penetration and cytotoxicity peptide data for training classification models.

`data/peptide_variable_descriptions.csv`

`data/mb_pilot_peptide_array_ml_input.csv`

`data/mb_second_peptide_array_ml_input.csv`

`data/cpp_ml_input.csv`

`data/tx_ml_input.csv`

1.2 Machine learning cross-validation results

A nested cross-validation framework was applied in this study, where the inner loop cross-validation is used to select the best performing subset of models, and the outer loop cross-validation is used estimate generalization performance. The `cv_res_statistical_testing.csv` files in the subfolders `outer_1` through `outer_10` correspond to each inner loop cross-validation fold, and in subfolder `whole_data_set` corresponds to the final outer loop cross-validation results. Models were scored and ranked based on multiple metrics. For regression the metrics were mean absolute error, root mean squared error and coefficient of determination (R^2). For classification the metrics were log loss, Matthews correlation coefficient, F_1 (harmonic mean of precision and recall), and balanced accuracy. The cross-metric rank was determined by summing the ranks of the individual metrics. Multiple statistical tests were performed by comparing the metric scores ($n = 10$) of the best model to all other models. Adjusted p-values were reported. The best model and models with no significant difference in all metrics from the best model were included in the files. The numbers of those competitive models were indicated in the parentheses below. The files containing grid search model parameters were also provided for melanin binding, cell-penetration, and cytotoxicity models.

1.2.1 Melanin binding models

`other_data/melanin_binding/neural_network_grid_params.csv`

`other_data/melanin_binding/gbm_grid_params.csv`

`other_data/melanin_binding/xgboost_grid_params.csv`

`other_data/melanin_binding/outer_1/cv_res_statistical_testing.csv` (2 competitive models)

`other_data/melanin_binding/outer_2/cv_res_statistical_testing.csv` (50 competitive models)

`other_data/melanin_binding/outer_3/cv_res_statistical_testing.csv` (15 competitive models)

`other_data/melanin_binding/outer_4/cv_res_statistical_testing.csv` (43 competitive models)

`other_data/melanin_binding/outer_5/cv_res_statistical_testing.csv` (41 competitive models)

`other_data/melanin_binding/outer_6/cv_res_statistical_testing.csv` (49 competitive models)

`other_data/melanin_binding/outer_7/cv_res_statistical_testing.csv` (19 competitive models)

`other_data/melanin_binding/outer_8/cv_res_statistical_testing.csv` (33 competitive models)

`other_data/melanin_binding/outer_9/cv_res_statistical_testing.csv` (32 competitive models)

`other_data/melanin_binding/outer_10/cv_res_statistical_testing.csv` (26 competitive models)

`other_data/melanin_binding/whole_data_set/cv_res_statistical_testing.csv` (31 competitive models)

1.2.2 Cell-penetration models

other_data/cell_penetration/neural_network_grid_params.csv

other_data/cell_penetration/gbm_grid_params.csv

other_data/cell_penetration/xgboost_grid_params.csv

other_data/cell_penetration/outer_1/cv_res_statistical_testing.csv (272 competitive models)

other_data/cell_penetration/outer_2/cv_res_statistical_testing.csv (227 competitive models)

other_data/cell_penetration/outer_3/cv_res_statistical_testing.csv (277 competitive models)

other_data/cell_penetration/outer_4/cv_res_statistical_testing.csv (303 competitive models)

other_data/cell_penetration/outer_5/cv_res_statistical_testing.csv (300 competitive models)

other_data/cell_penetration/outer_6/cv_res_statistical_testing.csv (303 competitive models)

other_data/cell_penetration/outer_7/cv_res_statistical_testing.csv (304 competitive models)

other_data/cell_penetration/outer_8/cv_res_statistical_testing.csv (303 competitive models)

other_data/cell_penetration/outer_9/cv_res_statistical_testing.csv (122 competitive models)

other_data/cell_penetration/outer_10/cv_res_statistical_testing.csv (304 competitive models)

other_data/cell_penetration/whole_data_set/cv_res_statistical_testing.csv (300 competitive models)

1.2.3 Cytotoxicity models

other_data/toxicity/neural_network_grid_params.csv

other_data/toxicity/gbm_grid_params.csv

other_data/toxicity/xgboost_grid_params.csv

other_data/toxicity/outer_1/cv_res_statistical_testing.csv (193 competitive models)

other_data/toxicity/outer_2/cv_res_statistical_testing.csv (49 competitive models)

other_data/toxicity/outer_3/cv_res_statistical_testing.csv (194 competitive models)

other_data/toxicity/outer_4/cv_res_statistical_testing.csv (74 competitive models)

other_data/toxicity/outer_5/cv_res_statistical_testing.csv (180 competitive models)

other_data/toxicity/outer_6/cv_res_statistical_testing.csv (197 competitive models)

other_data/toxicity/outer_7/cv_res_statistical_testing.csv (159 competitive models)

other_data/toxicity/outer_8/cv_res_statistical_testing.csv (179 competitive models)

other_data/toxicity/outer_9/cv_res_statistical_testing.csv (163 competitive models)

other_data/toxicity/outer_10/cv_res_statistical_testing.csv (153 competitive models)

other_data/toxicity/whole_data_set/cv_res_statistical_testing.csv (175 competitive models)

1.3 Adversarial control machine learning cross-validation results

To understand whether the whole machine learning procedure (including model selection) has learned meaningful relationships in the data sets, adversarial control models were trained on the data sets with the response variables randomly shuffled, and cross-validation results were reported for the inner loop iterations. Next, a best-performing model was selected in each inner loop cross-validation, and the generalize performance of these top one models were estimated in the outer loop cross-validation. There was no final predictive model trained on the whole data set in this experiment because it is unnecessary to use adversarial control models for future prediction, and thus there was no `whole_data_set` subfolders included. In the file list below, the values in the parentheses following the file names showed the numbers of competitive models filtered based on the statistical analyses of the model performance. See subsection 1.2 for detailed information. The metadata files containing the grid search model parameters were also included for reference.

1.3.1 Melanin binding adversarial control models

`other_data/melanin_binding_adversarial/neural_network_grid_params.csv`

`other_data/melanin_binding_adversarial/gbm_grid_params.csv`

`other_data/melanin_binding_adversarial/xgboost_grid_params.csv`

`other_data/melanin_binding_adversarial/outer_1/cv_res_statistical_testing.csv` (296 competitive models)

`other_data/melanin_binding_adversarial/outer_2/cv_res_statistical_testing.csv` (277 competitive models)

`other_data/melanin_binding_adversarial/outer_3/cv_res_statistical_testing.csv` (2 competitive models)

`other_data/melanin_binding_adversarial/outer_4/cv_res_statistical_testing.csv` (118 competitive models)

`other_data/melanin_binding_adversarial/outer_5/cv_res_statistical_testing.csv` (285 competitive models)

`other_data/melanin_binding_adversarial/outer_6/cv_res_statistical_testing.csv` (120 competitive models)

`other_data/melanin_binding_adversarial/outer_7/cv_res_statistical_testing.csv` (86 competitive models)

`other_data/melanin_binding_adversarial/outer_8/cv_res_statistical_testing.csv` (66 competitive models)

`other_data/melanin_binding_adversarial/outer_9/cv_res_statistical_testing.csv` (53 competitive models)

`other_data/melanin_binding_adversarial/outer_10/cv_res_statistical_testing.csv` (146 competitive models)

1.3.2 Cell-penetration adversarial control models

`other_data/cell_penetration_adversarial/neural_network_grid_params.csv`

`other_data/cell_penetration_adversarial/gbm_grid_params.csv`

`other_data/cell_penetration_adversarial/xgboost_grid_params.csv`

`other_data/cell_penetration_adversarial/outer_1/cv_res_statistical_testing.csv` (82 competitive models)

`other_data/cell_penetration_adversarial/outer_2/cv_res_statistical_testing.csv` (23 competitive models)

`other_data/cell_penetration_adversarial/outer_3/cv_res_statistical_testing.csv` (41 competitive models)

`other_data/cell_penetration_adversarial/outer_4/cv_res_statistical_testing.csv` (24 competitive models)

`other_data/cell_penetration_adversarial/outer_5/cv_res_statistical_testing.csv` (26 competitive models)

`other_data/cell_penetration_adversarial/outer_6/cv_res_statistical_testing.csv` (24 competitive models)

`other_data/cell_penetration_adversarial/outer_7/cv_res_statistical_testing.csv` (28 competitive models)

`other_data/cell_penetration_adversarial/outer_8/cv_res_statistical_testing.csv` (27 competitive models)

`other_data/cell_penetration_adversarial/outer_9/cv_res_statistical_testing.csv` (27 competitive models)

`other_data/cell_penetration_adversarial/outer_10/cv_res_statistical_testing.csv` (34 competitive models)

1.3.3 Cytotoxicity adversarial control models

`other_data/toxicity_adversarial/neural_network_grid_params.csv`

`other_data/toxicity_adversarial/gbm_grid_params.csv`

`other_data/toxicity_adversarial/xgboost_grid_params.csv`

`other_data/toxicity_adversarial/outer_1/cv_res_statistical_testing.csv` (141 competitive models)

`other_data/toxicity_penetration_adversarial/outer_2/cv_res_statistical_testing.csv` (126 competitive models)

`other_data/toxicity_penetration_adversarial/outer_3/cv_res_statistical_testing.csv` (118 competitive models)

`other_data/toxicity_penetration_adversarial/outer_4/cv_res_statistical_testing.csv` (130 competitive models)

`other_data/toxicity_penetration_adversarial/outer_5/cv_res_statistical_testing.csv` (80 competitive models)

`other_data/toxicity_penetration_adversarial/outer_6/cv_res_statistical_testing.csv` (78 competitive models)

`other_data/toxicity_penetration_adversarial/outer_7/cv_res_statistical_testing.csv` (131 competitive models)

`other_data/toxicity_penetration_adversarial/outer_8/cv_res_statistical_testing.csv` (131 competitive models)

`other_data/toxicity_penetration_adversarial/outer_9/cv_res_statistical_testing.csv` (136 competitive models)

`other_data/toxicity_penetration_adversarial/outer_10/cv_res_statistical_testing.csv` (127 competitive models)

2 Supplementary Figures

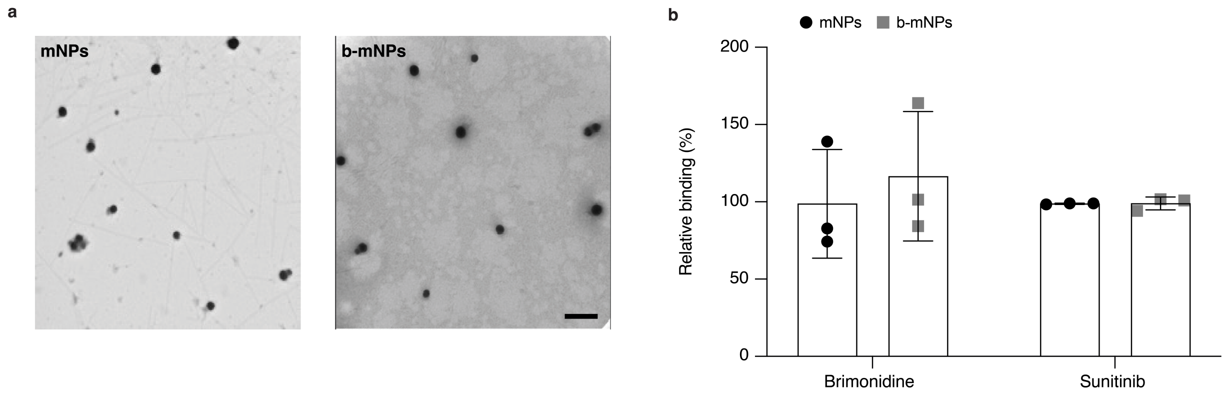

Supplementary Fig. 1 | Characterization of melanin nanoparticles (mNPs) and biotinylated-melanin nanoparticles (b-mNPs). a Representative transmission electron microscopes (TEM) images of mNPs and b-mNPs. The scale bar indicates 600 nm. **b** Relative binding of brimonidine tartrate and sunitinib malate to mNPs (black dots, $n = 3$ per drug group) and b-mNPs (gray squares, $n = 3$ per drug group). Data are shown as mean ± SD. No significant difference in relative binding was observed for either drug (Student's t-tests, two-tailed).

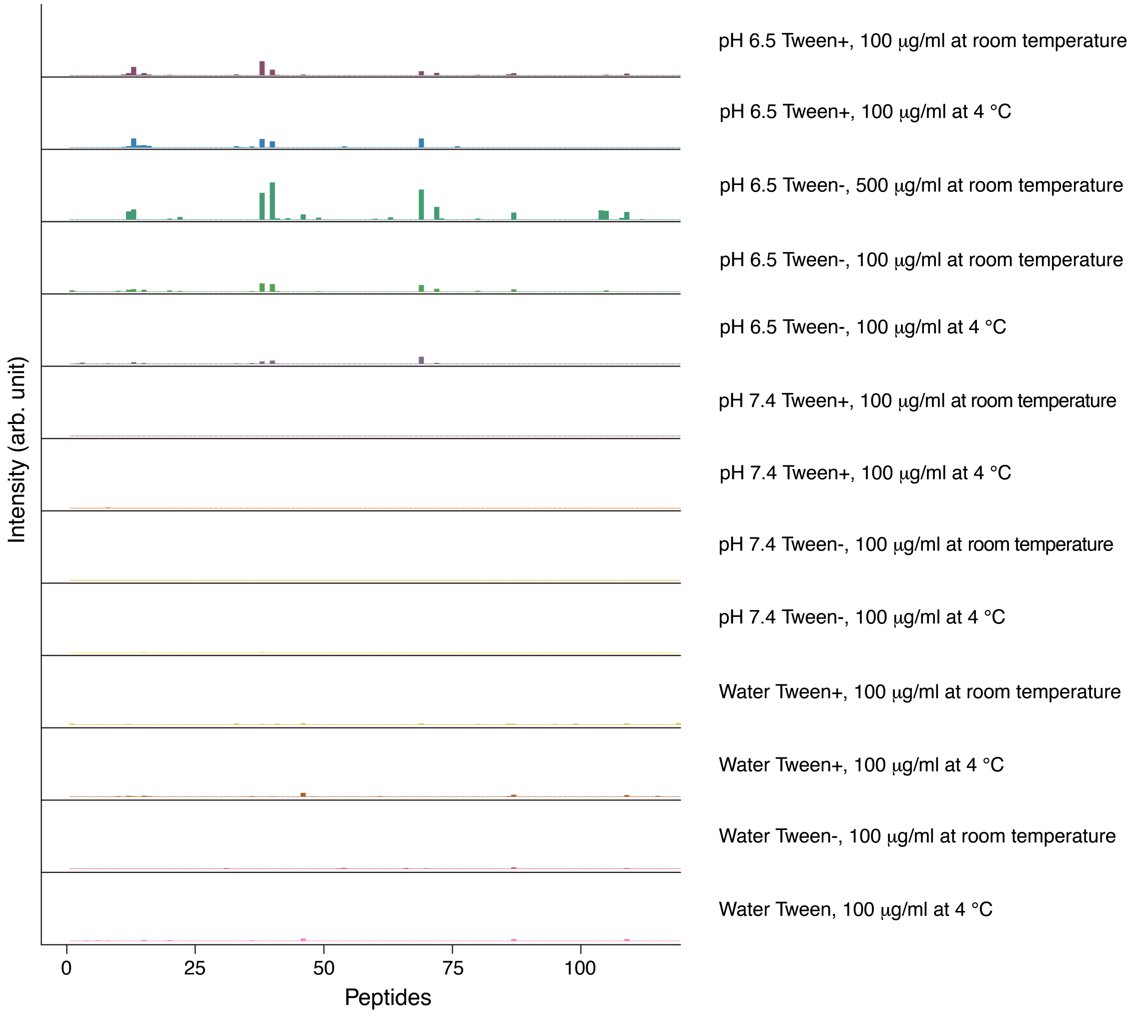

Supplementary Fig. 2 | Interaction profilings of b-mNPs against peptides in the pilot 119 microarray. Sparklines showing fluorescence intensities in varying washing buffer conditions (see **Methods** in the manuscript), plotted on the same scale in arbitrary unit (arb.unit). The first 16 peptides are positive control peptides, and the remaining are 103 random peptides.

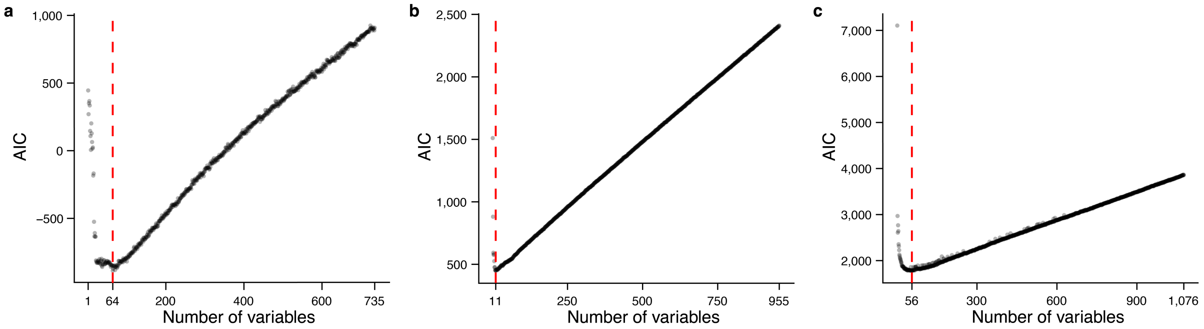

Supplementary Fig. 3 | Variable reduction of peptide data sets with random forests. a–c show performances of random forest models (black dots) trained on variable subsets ranked using permutation-based variable importance values for melanin binding (**a**), cell-penetration (**b**), and cytotoxicity (**c**) data sets. Akaike information criterion (AIC), a metric that penalizes complex models, was calculated for all models. The red dashed lines indicate the number of variables used in subsequent analyses.

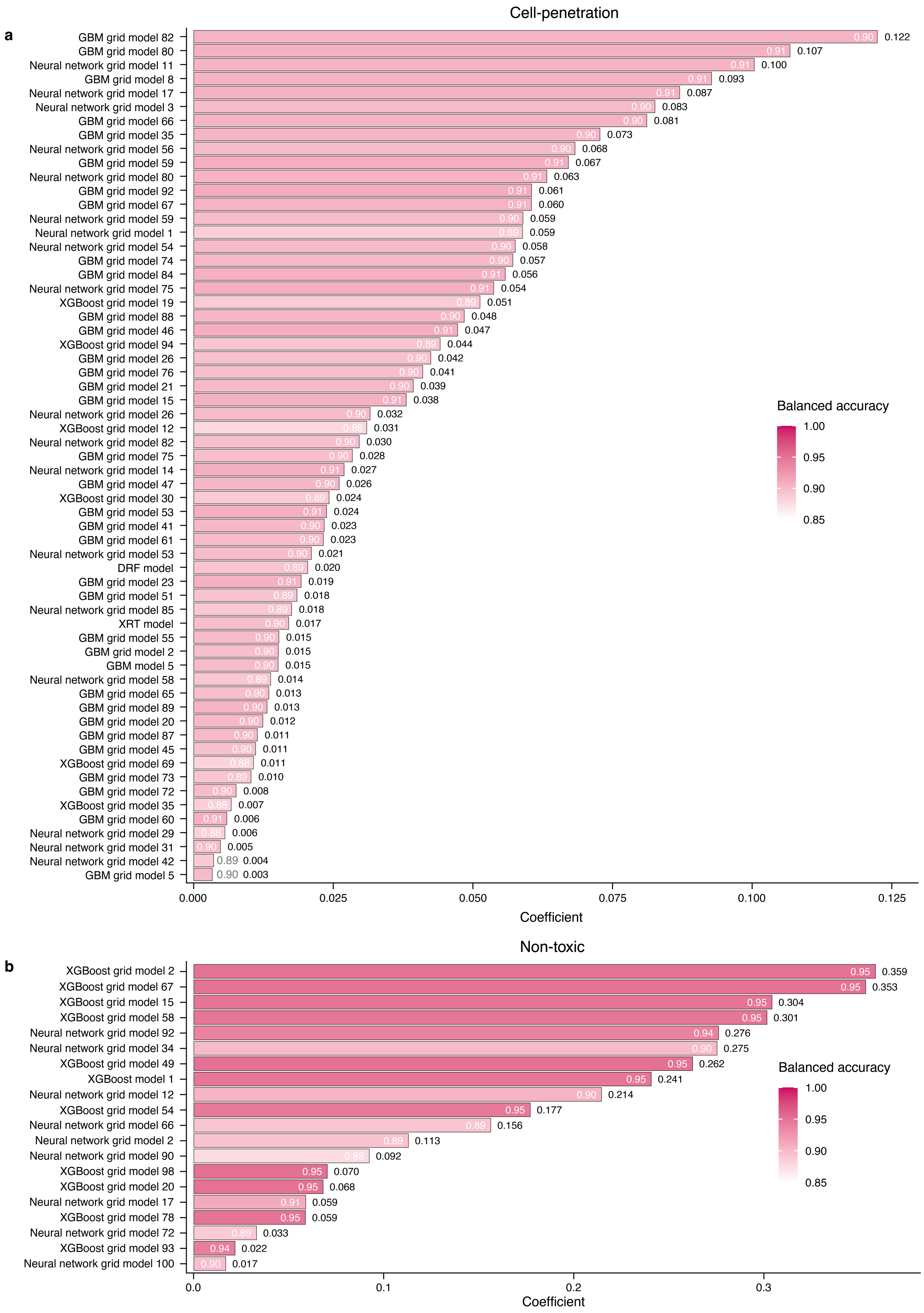

Supplementary Fig. 4 | Base model coefficients in final super learners. **a** Cell-penetration. **b** Cytotoxicity (with non-toxic peptides labeled as positive samples). Balanced accuracy is denoted with color and conveyed as white text on the bars or gray text adjacent bars. Values at the bar ends indicate base model coefficients. See **Methods** in the manuscript and **Supplementary Notes** (section 1) for more details regarding base model hyperparameters.

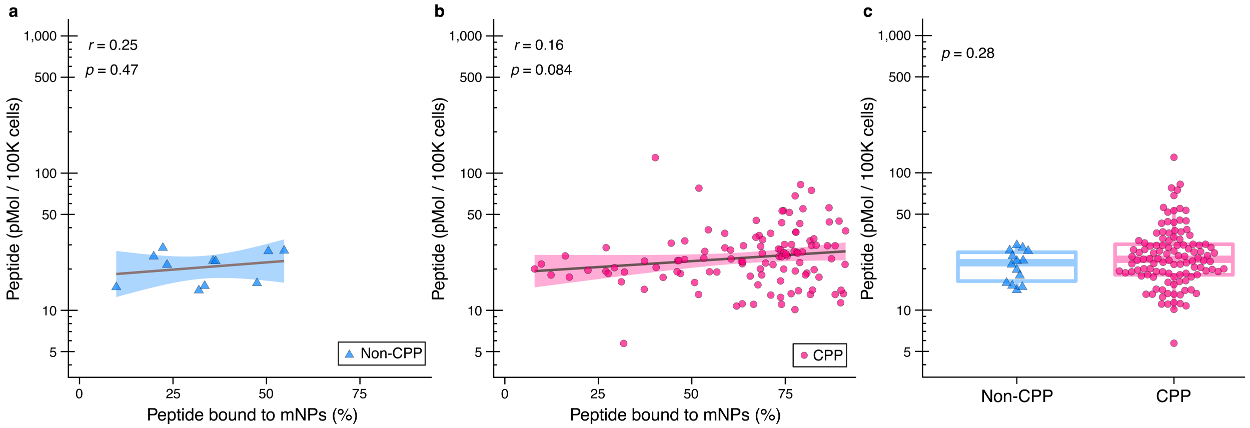

Supplementary Fig. 5 | Comparison of melanin binding and cell-penetration of candidate peptides in non-induced ARPE-19 cells. a, b Cyan triangles denote non-cell-penetrating peptides (non-CPP), and magenta dots represent cell-penetrating peptides (CPP). The x-axes indicate melanin binding measured from the mNP assay ($n = 4$), and the y-axes indicate intracellular concentration measured from the cell uptake assay with non-induced ARPE-19 cells ($n = 3$). Black linear trend lines indicate the Pearson correlation relationships, and the shaded areas convey 95% confidence intervals. The correlation coefficient and the corresponding p-values (two-tailed) are shown. **c** Intracellular concentrations of CPP ($n = 113$) and non-CPP ($n = 14$). Box plot indicates median (middle line), 25th and 75th percentiles (box), and the 1.5 × interquartile range (whiskers). The p-value was determined using a Mann–Whitney U test (two-tailed).

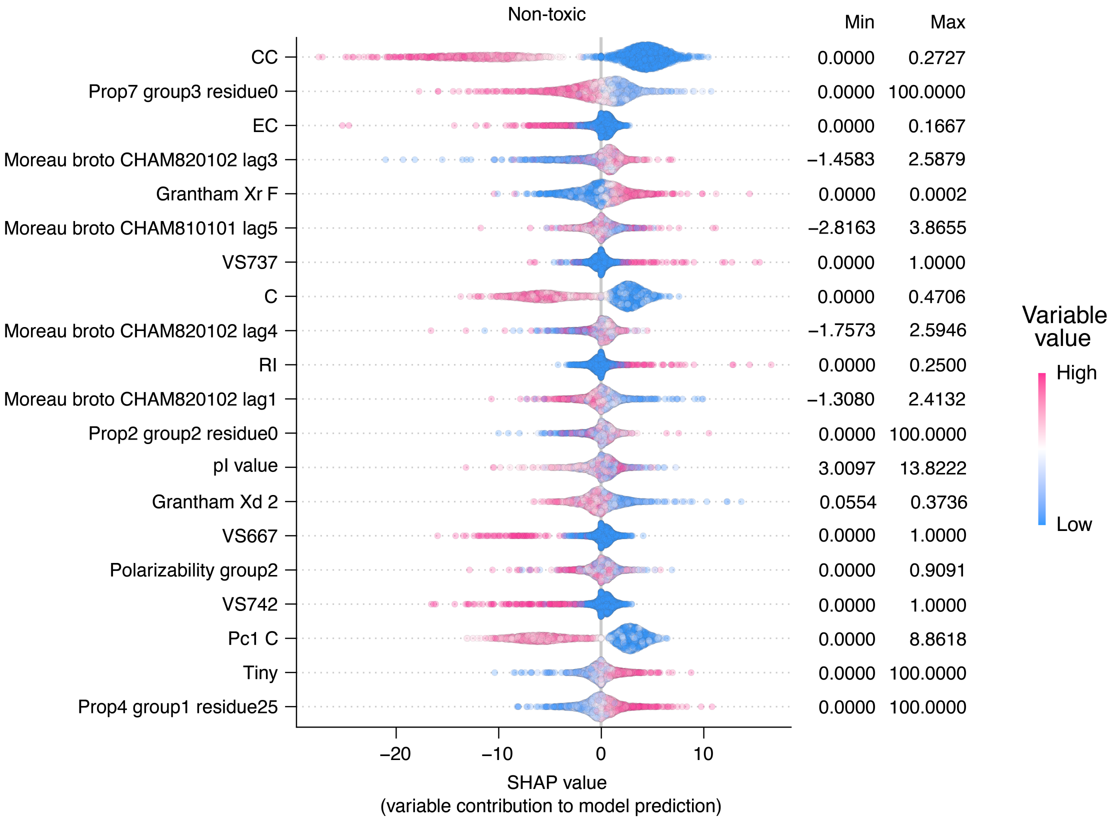

Supplementary Fig. 6 | Cytotoxicity model interpretation. Shapley additive explanation (SHAP) values for the top 20 variables ranked based on the SHAP value range. Dots represent peptides, and color indicates percentile ranks. The minimum and maximum variable values are listed on the right. See **Supplementary Data 8** for detailed variable descriptions.

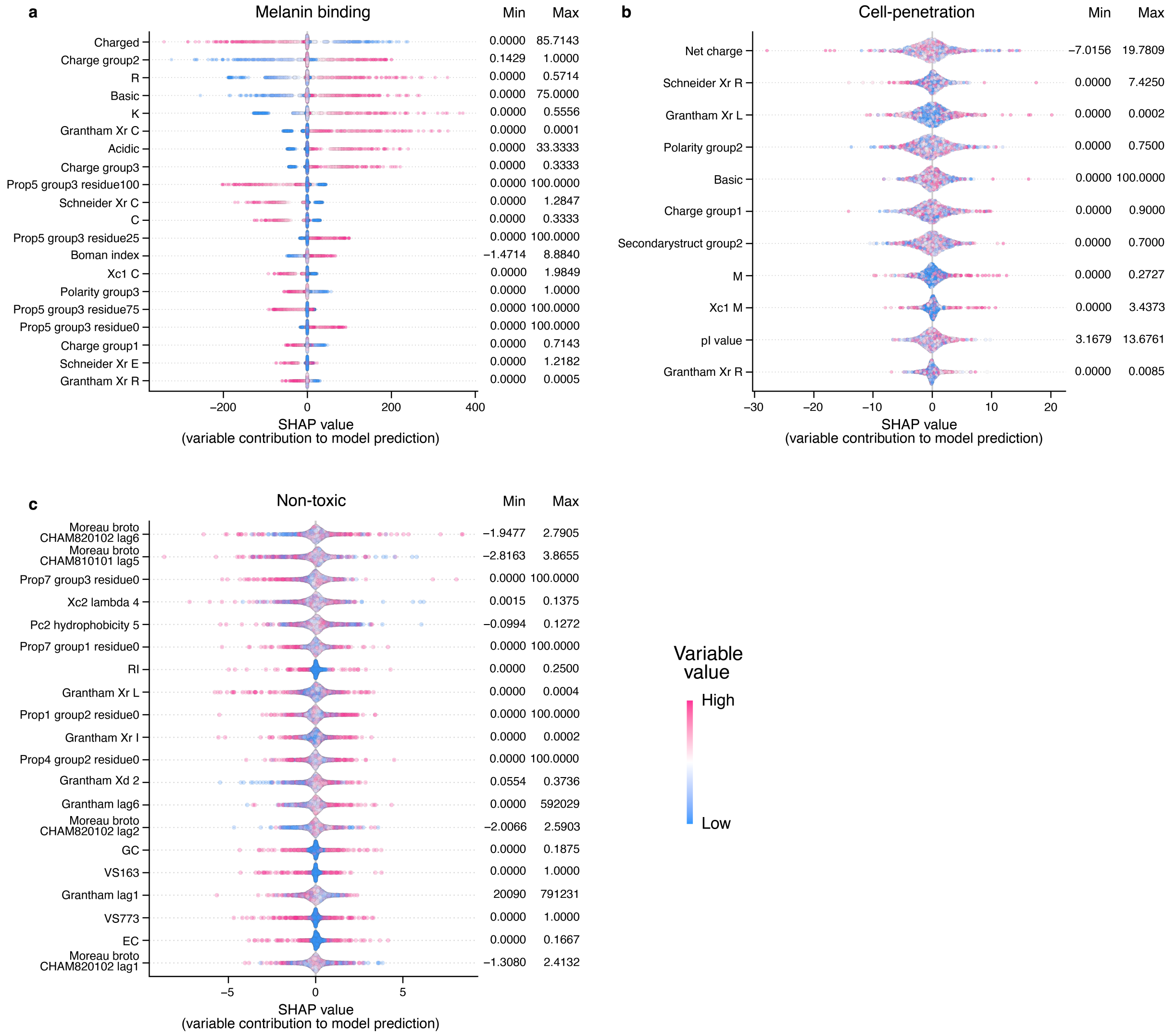

Supplementary Fig. 7 | Variable contributions to the prediction of the adversarial models. Top 20 important variables contributing to (**a**) melanin binding, (**b**) cell-penetration, and (**c**) cytotoxicity adversarial models. The variables were ranked based on the range of the SHAP values. Dots represent peptide samples. The color gradient shows the values of the corresponding variables, calculated as percent ranks. The minimum and maximum variables are shown on the right of each subfigure. See **Supplementary Data 8** for more about variable descriptions.

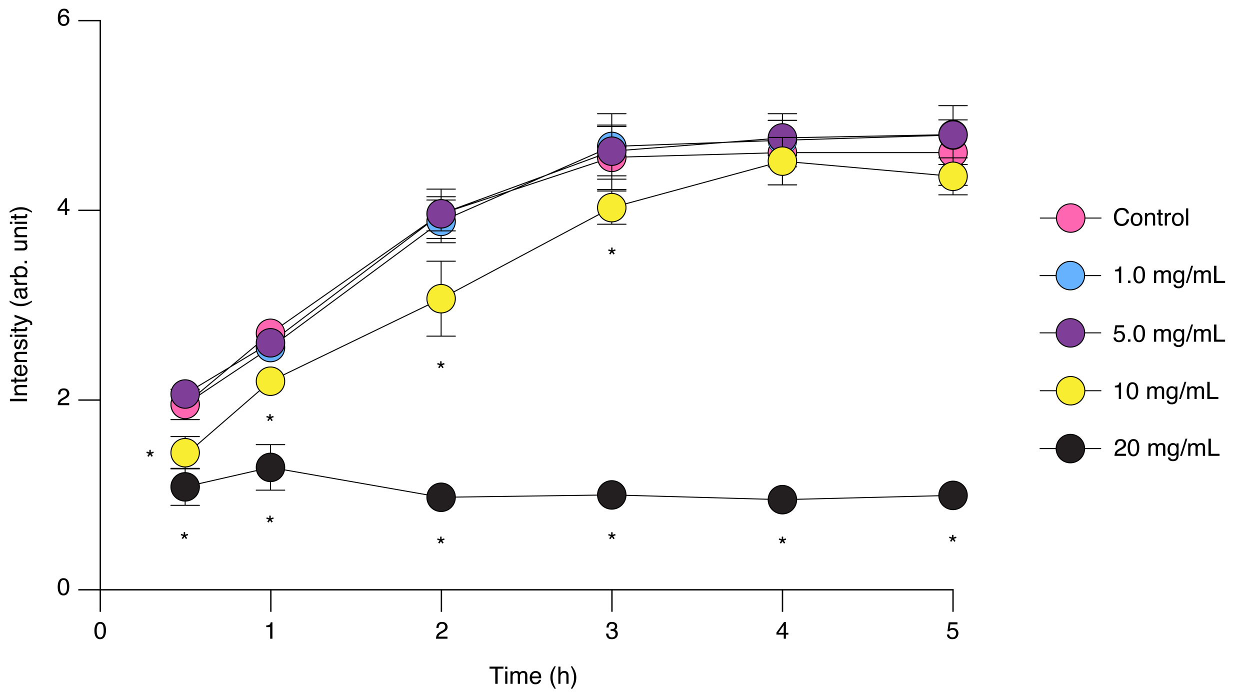

Supplementary Fig. 8 | Cytotoxicity validation of the HR97 peptide. Cell viability assay of the HR97 peptide. ARPE-19 cells were incubated with varying concentrations of HR97 for 12 h, and the cell viability was measured with the PrestoBlueTM HS cell viability system at 0.5, 1, 2, 3, 4, and 5 h after reagent addition ($n = 5$ per group). Data are presented as mean ± SD. HR97 concentration groups (1.0 mg/mL, cyan; 5.0 mg/mL, purple; 10 mg/mL, yellow; 20 mg/mL, black) were compared to the control group (magenta) with Student's t-tests (two-tailed). $*$ denotes $p < 0.05$. Adjusted p-values for 10 mg/mL vs. control at hours 0.5, 1, 2, and 3 were respectively 1.36×10^{-3}, 8.66×10^{-5}, 3.92×10^{-3}, and 1.73×10^{-2}; and those for 20 mg/mL vs. control at hours from 0.5 to 5 were 8.66×10^{-5}, 6.48×10^{-6}, 4.64×10^{-8}, 4.64×10^{-8}, 4.64×10^{-8}, and 4.80×10^{-8}, respectively.

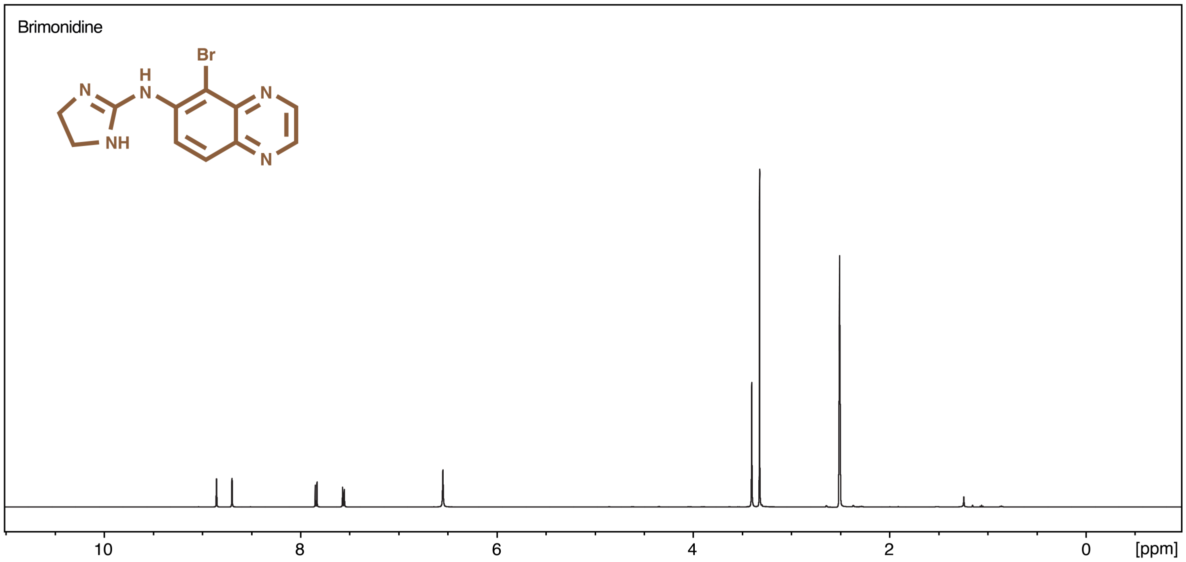

Supplementary Fig. 9 | NMR spectrum of brimonidine. The prep-HPLC retention time (RT) of brimonidine was 5.1 min. The molecular structure of brimonidine is shown in the upper left corner. Peak location and associated information are ^{1}H NMR (500 MHz, DMSO-d$_6$) 8.84 (d, J = 5 Hz, 1H), 8.68 (d, J = 5 Hz, 1H), 7.83 (d, J = 10Hz, 1H), 7.55 (d, J = 10Hz, 1H) 6.54 (s, 2H), 3.40 (s, 4H).

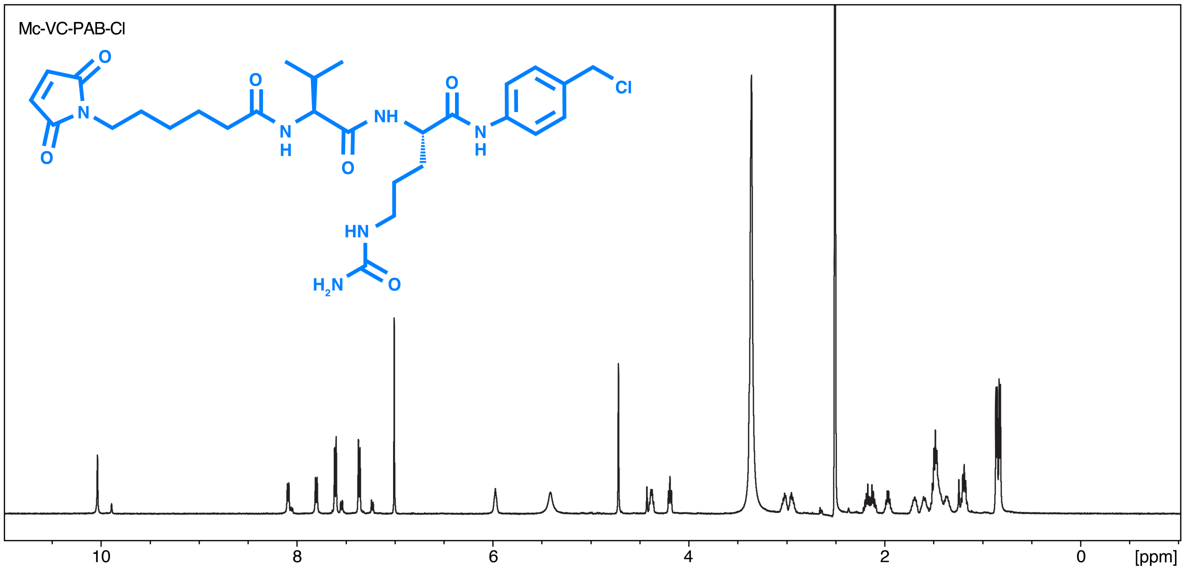

Supplementary Fig. 10 | NMR spectrum of Mc-VC-PAB-Cl (Maleimidocaproyl-L-valine-L-citrulline-p-aminobenzyl chloride). The prep-HPLC RT of Mc-VC-PAB-Cl was 11.4 min. The molecular structure of Mc-VC-PAB-Cl is shown in the upper left corner. Peak location and associated information of Mc-VC-PAB-Cl are ^{1}H NMR (500 MHz, DMSO-d_6) 10.03 (s, 1H), 8.08 (d, J = 7 Hz, 1H), 7.80 (d, J = 8.5 Hz, 1H), 7.60 (d, J = 8 Hz, 2H), 7.36 (d, J = 8.5 Hz, 2H), 7.01 (s, 2H), 5.97 (bs, 1H), 5.41 (vbs, 1H), 4.71 (s, 2H), 4.38 (t, J = 7.5Hz, 1H), 4.18 (dd, J = 1, 8 Hz 1H), 3.06–2.89 (m, 2H), 2.21–2.08 (m, 2H), 1.99–1.92 (m, 1H), 1.75–1.65 (m, 1H), 1.52–1.42(m, 5H), 1.38–1.31 (m, 1H), 1.19 (pen, J = 7.5 Hz, 2H), 0.86 (d, J = 6.5 Hz, 3H) 0.85 (d, J = 7 Hz, 3H).

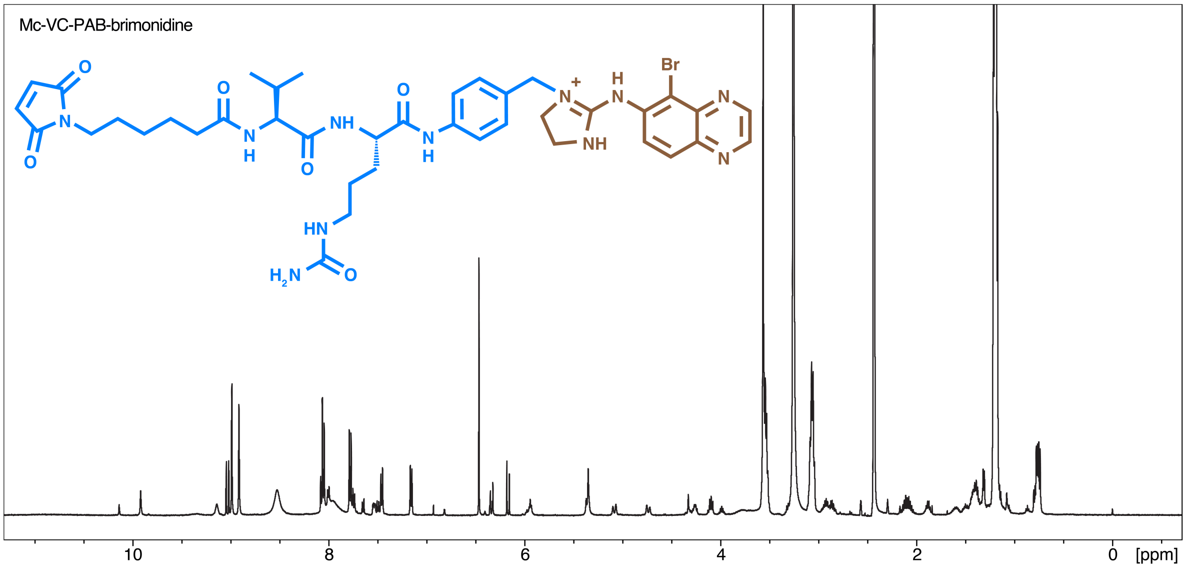

Supplementary Fig. 11 | NMR spectrum of Mc-VC-PAB-brimonidine. The prep-HPLC RT of Mc-VC-PAB-brimonidine was 9.8 min. The molecular structure of Mc-VC-PAB-brimonidine is shown in the upper left corner. Peak location and associated information are ^{1}H NMR (500 MHz, DMSO-d_6) 9.98 (s, 1H), 9.21 (bs, 1H), 9.11 (d, J = 1.5 Hz, 1H), 9.08 (d, J = 2 Hz, 1H) 9.05 (d, J = 1.5 Hz, 3H), 8.98 (s, 1H), 8.59 (bs, 5H), 8.15 (s, 1H), 8.12 (d, J = 9 Hz, 5H), 8.07(d, J = 7.5 Hz, 2H), 7.84 (d, J = 9Hz, 4H), 7.81 (d, J = 9 Hz, 1H), 7.71 (d, J = 8.5 Hz, 1H), 7.60 (dd, J = 4.5, 8.5 Hz, 1H), 7.52 (d, J = 8.5 Hz, 2H), 7.22 (d, J = 8.5 Hz, 2H), 6.53 (s, 3H), 6.40 (dd, J = 3.5, 27 Hz, 2H), 6.25 (s, 1H), 6.22 (s, 1H), 6.06–6.00 (m, 3H), 5.42 (d, J = 10.5 Hz, 5H), 5.15 (d, J = 14.5 Hz, 2H), 4.81 (d, J = 18.5 Hz, 2H), 4.33 (s, 1H), 4.25 (m, 2H), 4.13–4.07 (m, 2H), 4.01–3.96 (m, 1H), 2.96–2.82 (m, 4H), 2.17–2.03 (m, 4H), 1.87 (dd, J = 6.5 Hz, 2H), 1.64–1.56 (m, 2H), 1.53–1.48 (m, 2H), 1.44–1.36 (m, 4H), 1.32–1.31 (d, J = 6.5 Hz, 4H), 0.76 (d, J = 6.5 Hz, 3H), 0.74 (d, J = 6.5 Hz, 3H).

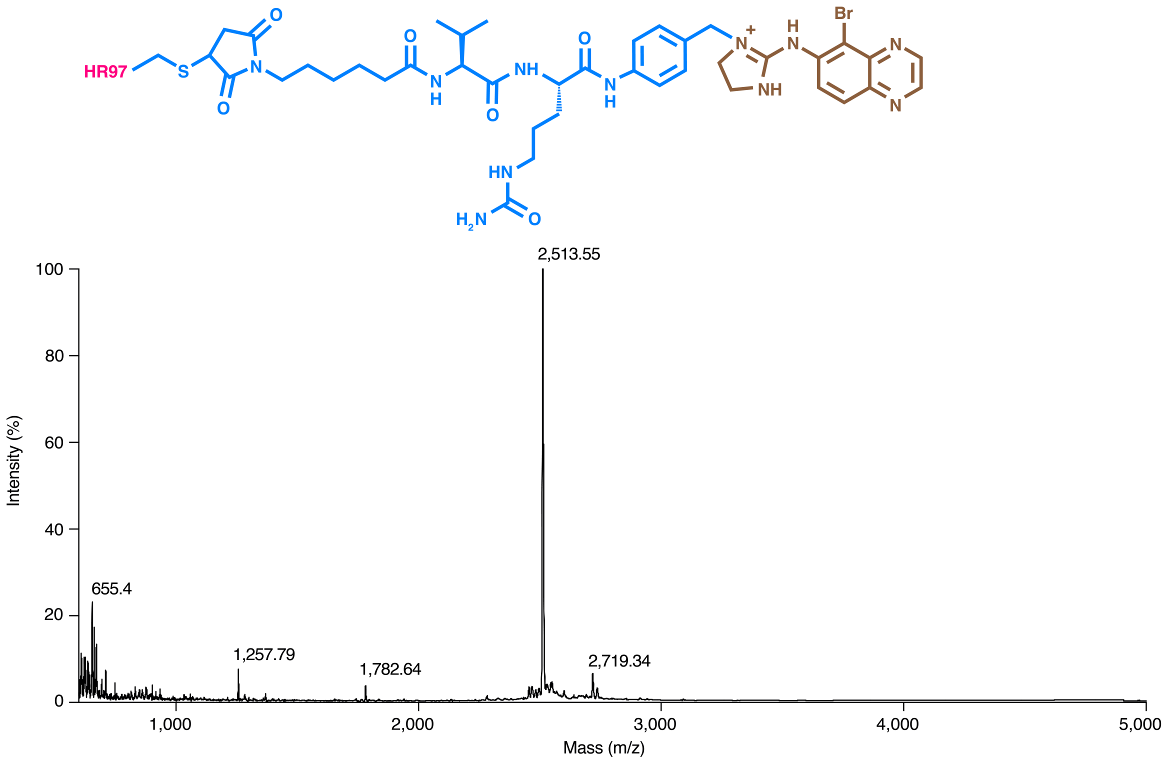

Supplementary Fig. 12 | MALDI-TOF spectrum of the HR97-brimonidine conjugate. The molecular structure of HR97-(quaternary-ammonium-linked)-brimonidine conjugate is shown in the upper left corner. The *m/z* calculated for $C_{103}H_{162}BN_{38}O_{20}S^{+}$ was 2,513.19, and 2,513.55 $[M - 5H^{+} + 5Na^{+} + K^{+}]$ was found.

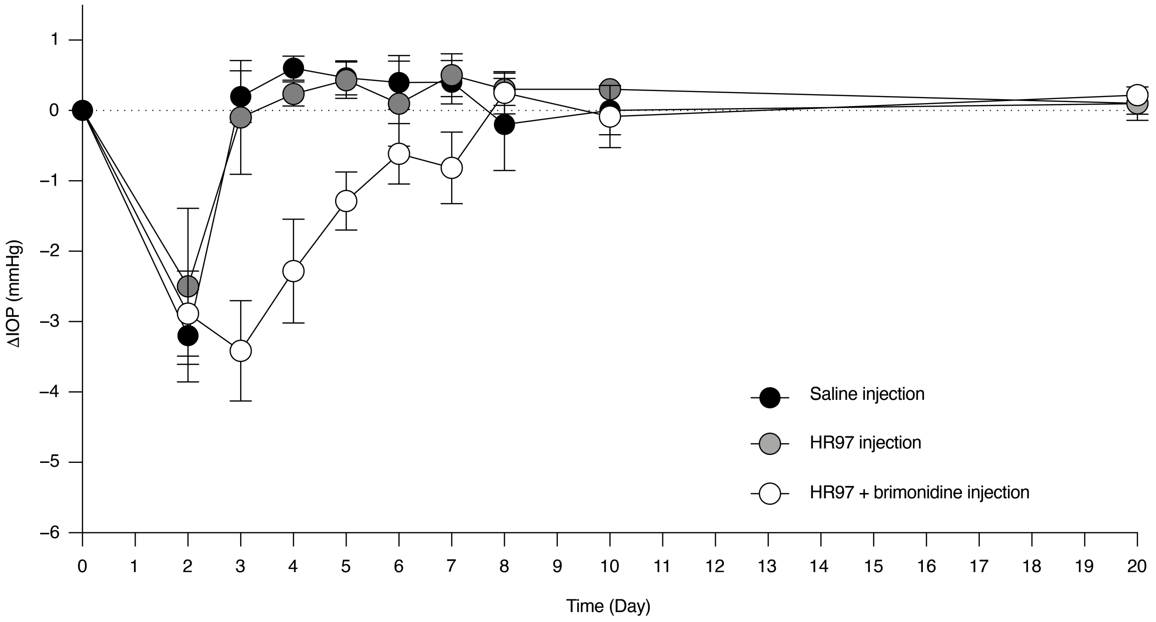

Supplementary Fig. 13 | Comparison of intraocular pressure (IOP) change from baseline. Line plot showing the IOP change from baseline (ΔIOP) after a single ICM injection of saline (black dots), HR97 (gray dots, equivalent to the amount of HR97 in HR97-brimonidine conjugate), and a physical mixture of HR97 and brimonidine tartrate in solution (white dots, HR97 + brimonidine, 200 μg brimonidine equivalent) in normotensive Dutch Belted rabbits (n = 5 per group). The IOP was measured every 1–2 days post-injection until measured value reached or exceeded baseline, and again at day 20 post-injection. Data are shown as mean ± SEM.

3 Supplementary Tables

Supplementary Table 1 | Cross-validation performance (mean ± SEM) of the melanin binding general and adversarial control models

Metric	General model	Adversarial control
Mean absolute error[a]	16.812 ± 0.166	32.857 ± 0.173
Root mean squared error[a]	22.782 ± 0.222	34.344 ± 0.226
Coefficient of determination (R^2)	0.543 ± 0.007	-0.038 ± 0.015

[a]Percent normalized values presented.

Supplementary Table 2 | Cross-validation performance (mean ± SEM) of the cell-penetration general and adversarial control models

Metric	General model	Adversarial control
Log loss	0.259 ± 0.017	0.715 ± 0.013
Matthews correlation coefficient	0.794 ± 0.014	-0.002 ± 0.054
F_1[a]	0.901 ± 0.005	0.522 ± 0.031
Balanced accuracy	0.897 ± 0.007	0.502 ± 0.028
Enrichment factor	2.081 ± 0.065	1.090 ± 0.159
BEDROC[b]	0.999 ± 0.000	0.529 ± 0.058

[a]Harmonic mean of precision and recall.

[b]Boltzmann-enhanced discrimination of receiver operating characteristic.

Supplementary Table 3 | Cross-validation performance (mean ± SEM) of the cytotoxicity general and adversarial control models

Metric	General model	Adversarial control
Log loss	0.172 ± 0.008	0.654 ± 0.005
Matthews correlation coefficient	0.879 ± 0.004	0.001 ± 0.012
F_1[a]	0.919 ± 0.002	0.047 ± 0.023
Balanced accuracy	0.947 ± 0.002	0.619 ± 0.035
Enrichment factor	1.519 ± 0.017	0.956 ± 0.057
BEDROC[b]	0.993 ± 0.005	0.620 ± 0.023

[a]Harmonic mean of precision and recall.

[b]Boltzmann-enhanced discrimination of receiver operating characteristic.

Supplementary Table 4 | Ocular grading 7 days after a single ICM injection of saline, HR97 (equivalent to the amount of HR97 in HR97-brimonidine conjugate), or a physical mixture of HR97 and brimonidine tartrate in solution (HR97 + brimonidine, 200 μg brimonidine equivalent) in Dutch Belted rabbits ($n = 5$ per group)

**Day 7**	**Saline**					**Peptide**					**Peptide+brimonidine mixture**				
Rabbit ID	275	261	262	263	264	265	269	270	271	272	273	274	266	267	268
Pupillary light reflex	0	0	0	0	0	0	0	0	0	0	0	0	0	0	0
Conjunctival hypermia	0	0	0	0	0	0	0	0	0	0	0	0	0	0	0
Conjunctival swelling	0	0	0	0	0	0	0	0	0	0	0	0	0	0	0
Conjunctival discharge	0	0	0	0	0	0	0	0	0	0	0	0	0	0	0
Corneal opacity (severity)	0	0	0	0	0	0	0	0	0	0	0	0	0	0	0
Corneal opacity (area)	0	0	0	0	0	0	0	0	0	0	0	0	0	0	0
Corneal vascularization	0	0	0	0	0	0	0	0	0	0	0	0	0	0	0
Aqueous flare	0	0	0	0	0	0	0	0	0	0	0	0	0	0	0
Anterior chamber cells	0	0	0	0	0	0	0	0	0	0	0	0	0	0	0
Iris involvement	0	0	0	0	0	0	0	0	0	0	0	0	0	0	0
Anterior vitreous cells	0	0	0	0	0	0	0	0	0	0	0	0	0	0	0
Fluorescein staining (severity)	0	0	0	0	0	0	0	0	0	0	0	0	0	0	0
Fluorescein staining (area)	0	0	0	0	0	0	0	0	0	0	0	0	0	0	0
Eyelid discharge	0	0	0	0	0	0	0	0	0	0	0	0	0	0	0
Eyelid swelling	0	0	0	0	0	0	0	0	0	0	0	0	0	0	0
Eyelid vascularity	0	0	0	0	0	0	0	0	0	0	0	0	0	0	0
Meibomian gland function	0	0	0	0	0	0	0	0	0	0	0	0	0	0	0

All values are zero.

Supplementary Table 5 | Ocular grading 14 days after a single ICM injection of saline, HR97 (equivalent to the amount of HR97 in HR97-brimonidine conjugate), or a physical mixture of HR97 and brimonidine tartrate in solution (HR97 + brimonidine, 200 μg brimonidine equivalent) in Dutch Belted rabbits ($n = 5$ per group)

**Day 14**	**Saline**					**Peptide**					**Peptide+brimonidine mixture**				
Rabbit ID	275	261	262	263	264	265	269	270	271	272	273	274	266	267	268
Pupillary light reflex	0	0	0	0	0	0	0	0	0	0	0	0	0	0	0
Conjunctival hypermia	0	0	0	0	0	0	0	0	0	0	0	0	0	0	0
Conjunctival swelling	0	0	0	0	0	0	0	0	0	0	0	0	0	0	0
Conjunctival discharge	0	0	0	0	0	0	0	0	0	0	0	0	0	0	0
Corneal opacity (severity)	0	0	0	0	0	0	0	0	0	0	0	0	0	0	0
Corneal opacity (area)	0	0	0	0	0	0	0	0	0	0	0	0	0	0	0
Corneal vascularization	0	0	0	0	0	0	0	0	0	0	0	0	0	0	0
Aqueous flare	0	0	0	0	0	0	0	0	0	0	0	0	0	0	0
Anterior chamber cells	0	0	0	0	0	0	0	0	0	0	0	0	0	0	0
Iris involvement	0	0	0	0	0	0	0	0	0	0	0	0	0	0	0
Anterior vitreous cells	0	0	0	0	0	0	0	0	0	0	0	0	0	0	0
Fluorescein staining (severity)	0	0	0	0	0	0	0	0	**1**	0	0	0	0	0	0
Fluorescein staining (area)	0	0	0	0	0	0	0	0	**1**	0	0	0	0	0	0
Eyelid discharge	0	0	0	0	0	0	0	0	0	0	0	0	0	0	0
Eyelid swelling	0	0	0	0	0	0	0	0	0	0	0	0	0	0	0
Eyelid vascularity	0	0	0	0	0	0	0	0	0	0	0	0	0	0	0
Meibomian gland function	0	0	0	0	0	0	0	0	0	0	0	0	0	0	0

All non-zero values are noted in bold.

Supplementary Table 6 | Ocular grading 21 days after a single ICM injection of saline, HR97 (equivalent to the amount of HR97 in HR97-brimonidine conjugate), or a physical mixture of HR97 and brimonidine tartrate in solution (HR97 + brimonidine, 200 μg brimonidine equivalent) in Dutch Belted rabbits ($n = 5$ per group)

**Day 21**	**Saline**					**Peptide**					**Peptide+brimonidine mixture**				
Rabbit ID	275	261	262	263	264	265	269	270	271	272	273	274	266	267	268
Pupillary light reflex	0	0	0	0	0	0	0	0	0	0	0	0	0	0	0
Conjunctival hypermia	0	0	0	0	0	0	0	0	0	0	0	0	0	0	0
Conjunctival swelling	0	0	0	0	0	0	0	0	0	0	0	0	0	0	0
Conjunctival discharge	0	0	0	0	0	0	0	0	0	0	0	0	0	0	0
Corneal opacity (severity)	0	0	0	0	0	0	0	0	0	0	0	0	0	0	0
Corneal opacity (area)	0	0	0	0	0	0	0	0	0	0	0	0	0	0	0
Corneal vascularization	0	0	0	0	0	0	0	0	0	0	0	0	0	0	0
Aqueous flare	0	0	0	0	0	0	0	0	0	0	0	0	0	0	0
Anterior chamber cells	0	0	0	0	0	0	0	0	0	0	0	0	0	0	0
Iris involvement	0	0	0	0	0	0	0	0	0	0	0	0	0	0	0
Anterior vitreous cells	0	0	0	0	0	0	0	0	0	0	0	0	0	0	0
Fluorescein staining (severity)	0	0	0	0	**1**	0	0	0	0	0	0	0	0	0	0
Fluorescein staining (area)	0	0	0	0	**1**	0	0	0	0	0	0	0	0	0	0
Eyelid discharge	0	0	0	0	0	0	0	0	0	0	0	0	0	0	0
Eyelid swelling	0	0	0	0	0	0	0	0	0	0	0	0	0	0	0
Eyelid vascularity	0	0	0	0	0	0	0	0	0	0	0	0	0	0	0
Meibomian gland function	0	0	0	0	0	0	0	0	0	0	0	0	0	0	0

All non-zero values are noted in bold.

Supplementary Table 7 | Ocular grading 28 days after a single ICM injection of saline, HR97 (equivalent to the amount of HR97 in HR97-brimonidine conjugate), or a physical mixture of HR97 and brimonidine tartrate in solution (HR97 + brimonidine, 200 μg brimonidine equivalent) in Dutch Belted rabbits ($n = 5$ per group)

**Day 28**	**Saline**					**Peptide**					**Peptide+brimonidine mixture**				
Rabbit ID	275	261	262	263	264	265	269	270	271	272	273	274	266	267	268
Pupillary light reflex	0	0	0	0	0	0	0	0	0	0	0	0	0	0	0
Conjunctival hypermia	0	0	0	0	0	0	0	0	0	0	0	0	0	0	0
Conjunctival swelling	0	0	0	0	0	0	0	0	0	0	0	0	0	0	0
Conjunctival discharge	0	0	0	0	0	0	0	0	0	0	0	0	0	0	0
Corneal opacity (severity)	0	0	0	0	0	0	0	0	0	0	0	0	0	0	0
Corneal opacity (area)	0	0	0	0	0	0	0	0	0	0	0	0	0	0	0
Corneal vascularization	0	0	0	0	0	0	0	0	0	0	0	0	0	0	0
Aqueous flare	0	0	0	0	0	0	0	0	0	0	0	0	0	0	0
Anterior chamber cells	0	0	0	0	0	0	0	0	0	0	0	0	0	0	0
Iris involvement	0	0	0	0	0	0	0	0	0	0	0	0	0	0	0
Anterior vitreous cells	0	0	0	0	0	0	0	0	0	0	0	0	0	0	0
Fluorescein staining (severity)	0	0	0	0	0	0	0	0	0	0	0	0	0	0	0
Fluorescein staining (area)	0	0	0	0	0	0	0	0	0	0	0	0	0	0	0
Eyelid discharge	0	0	0	0	0	0	0	0	0	0	0	0	0	0	0
Eyelid swelling	0	0	0	0	0	0	0	0	0	0	0	0	0	0	0
Eyelid vascularity	0	0	0	0	0	0	0	0	0	0	0	0	0	0	0
Meibomian gland function	0	0	0	0	0	0	0	0	0	0	0	0	0	0	0

All values are zero.
