# Supplementary material for: Machine learning-driven multifunctional peptide engineering for sustained ocular drug delivery
Source: Nat Commun. 2023 May 2;14:2509. doi: 10.1038/s41467-023-38056-w (PMC10154330; doi:10.1038/s41467-023-38056-w)
Supplement: Supplementary file 3 — Description of Additional Supplementary Files [file 41467_2023_38056_MOESM3_ESM.pdf]

Description of Additional Supplementary Files

Supplementary Data 1: Melanin binding pilot peptide microarray machine learning input data set.

Supplementary Data 2: Melanin binding second peptide microarray machine learning input data set.

Supplementary Data 3: Melanin binding cross-validation statistical analysis results.

Supplementary Data 4: Cell-penetration peptide machine learning input data set. Supplementary Data 5: Cytotoxicity peptide machine learning input data set.

Supplementary Data 6: Cell-penetration cross-validation statistical analysis results.

Supplementary Data 7: Cytotoxicity crossvalidation statistical analysis results.

Supplementary Data 8: Peptide variable descriptions.

Supplementary Data 9: NMR acquisition parameters.
