# Supplementary material for: Machine learning-driven multifunctional peptide engineering for sustained ocular drug delivery
Source: Nat Commun. 2023 May 2;14:2509. doi: 10.1038/s41467-023-38056-w (PMC10154330; doi:10.1038/s41467-023-38056-w)
Supplement: Supplementary file 13 — Reporting Summary [file 41467_2023_38056_MOESM13_ESM.pdf]

nature portfolio

Corresponding author(s): Laura M. Ensign and Michael P. Cummings

Last updated by author(s): Apr 5, 2023

Reporting Summary

Nature Portfolio wishes to improve the reproducibility of the work that we publish. This form provides structure for consistency and transparency in reporting. For further information on Nature Portfolio policies, see our Editorial Policies and the Editorial Policy Checklist.

Statistics

For all statistical analyses, confirm that the following items are present in the figure legend, table legend, main text, or Methods section.

n/a	Confirmed	
☐	☒	The exact sample size (*n*) for each experimental group/condition, given as a discrete number and unit of measurement
☐	☒	A statement on whether measurements were taken from distinct samples or whether the same sample was measured repeatedly
☐	☒	The statistical test(s) used AND whether they are one- or two-sided<br>*Only common tests should be described solely by name; describe more complex techniques in the Methods section.*
☐	☒	A description of all covariates tested
☐	☒	A description of any assumptions or corrections, such as tests of normality and adjustment for multiple comparisons
☐	☒	A full description of the statistical parameters including central tendency (e.g. means) or other basic estimates (e.g. regression coefficient) AND variation (e.g. standard deviation) or associated estimates of uncertainty (e.g. confidence intervals)
☐	☒	For null hypothesis testing, the test statistic (e.g. *F*, *t*, *r*) with confidence intervals, effect sizes, degrees of freedom and *P* value noted<br>*Give P values as exact values whenever suitable.*
☒	☐	For Bayesian analysis, information on the choice of priors and Markov chain Monte Carlo settings
☒	☐	For hierarchical and complex designs, identification of the appropriate level for tests and full reporting of outcomes
☐	☒	Estimates of effect sizes (e.g. Cohen's *d*, Pearson's *r*), indicating how they were calculated

Our web collection on statistics for biologists contains articles on many of the points above.

Software and code

Policy information about availability of computer code

Data collection	Cell-penetration peptides were from the SkipCPP-Pred (Wei et al., BMC Genomics, 18(7), 742.; https://bmcgenomics.biomedcentral.com/articles/10.1186/s12864-017-4128-1) and cytotoxicity peptides were collected from ToxinPred (Gupta et al., PLoS One, 8(9), e73957.; http://crdd.osdd.net/raghava/toxinpred/). Peptide variables were computed using R packages Peptides, version 2.4.4 and protr, version 1.6-2.
Data analysis	Peptide microarray image analysis was conducted using PepSlide® Analyzer, version 1.4. Random forest algorithm was performed using R packages randomForest, version 4.7-1.1 and ranger, version 0.14.1. Super learners, neural networks, gradient boosting machines (GBM), extreme gradient boosting (XGBoost), generalized linear model (GLM), (distributed) random forest (DRF), and extremely randomized trees (XRT) were built using the R interface of H2O.ai, version 3.38.0.2. Shapley additive explanations (SHAP) analysis was conducted using the Python package SHAP, version 0.41.0, with machine learning models imported using the Python interface of H2O.ai, version 3.38.0.2. t-SNE analysis was performed using the R package Rtsne, version 0.16. Absorbance/Spectrum data were collected via the manufacturer's provided soware (Gen5 v.1.1). NMR data were analyzed using Bruker TopSpin Software, version 4.1.0. Statistical analyses were conducted using GraphPad Prism 9 or R version 4.2.2 (2022-10-31). All code and the implemented machine learning pipeline has been deposited in the Digital Repository at the University of Maryland (DRUM), https://doi.org/10.13016/0jck-hnnv.

For manuscripts utilizing custom algorithms or software that are central to the research but not yet described in published literature, software must be made available to editors and reviewers. We strongly encourage code deposition in a community repository (e.g. GitHub). See the Nature Portfolio guidelines for submitting code & software for further information.

Data

Policy information about availability of data

All manuscripts must include a data availability statement. This statement should provide the following information, where applicable:

- Accession codes, unique identifiers, or web links for publicly available datasets
- A description of any restrictions on data availability
- For clinical datasets or third party data, please ensure that the statement adheres to our policy

Data sets, source data for figure generation, and all final property models are available as compressed files deposited in the Digital Repository at the University of Maryland (DRUM), https://doi.org/10.13016/0jck-hnnv. Cell-penetration and cytotoxicity data sets are available on the SkipCPP-Pred (Wei et al., BMC Genomics, 18(7), 742.; https://bmcgenomics.biomedcentral.com/articles/10.1186/s12864-017-4128-1) and ToxinPred (Gupta et al., PLoS One, 8(9), e73957.; http://crdd.osdd.net/raghava/toxinpred/) websites, respectively. Source data are provided with this paper.

Human research participants

Policy information about studies involving human research participants and Sex and Gender in Research.

Reporting on sex and gender	Two pairs of eyes were obtained from two male donors.
Population characteristics	Two pairs of human donor eyes were obtained from the Lions Gift of Sight under protocol IRB00056984 approved by the Johns Hopkins University School of Medicine Institutional Review Board. Both donors were male and with age of 82 & 62. The post-mortem times ranged from 35–40 h.
Recruitment	Donors were recruited by Lions Gift of Sight, and eye specimens were purchased for research use.
Ethics oversight	The protocol IRB00056984 was approved by the Johns Hopkins University School of Medicine Institutional Review Board.

Note that full information on the approval of the study protocol must also be provided in the manuscript.

Field-specific reporting

Please select the one below that is the best fit for your research. If you are not sure, read the appropriate sections before making your selection.

☒ Life sciences ☐ Behavioural & social sciences ☐ Ecological, evolutionary & environmental sciences

For a reference copy of the document with all sections, see nature.com/documents/nr-reporting-summary-flat.pdf

Life sciences study design

All studies must disclose on these points even when the disclosure is negative.

Sample size	For peptide microarrays, the PepPerPrint company protocol includes removing spot-to-spot deviations larger than 40% between replicates. Based on the low observed variability between peptide microarray replicates, the company determined that 2 replicates was sufficient. Sample sizes for in vitro analytical assays, such as the in vitro melanin binding assay, cathepsin assay, cell uptake assay, and stability assay, were set at n = 3 due to the standardized sample preparation and well-characterized starting materials. Previous research supports this sample size as sufficient to detect meaningful differences for in vitro analytical samples (Kim et al., Drug Delivery and Translational Research, 12(4), 826–837.; Staben et al., Nature Chemistry, 8(12), 1112–1119.) Thus, no statistical calculations were made to determine the sample size. For IOP measurements in normotensive rabbits, previous group research with Dutch Belted rabbits and a clinically meaningful difference of 2 mmHg has shown differences between control and brimonidine-treated groups of approximately 12 mmHg with a standard deviation of 2.51 mmHg. Calculation using 5% type 1 error and 80% power supports a group size of n = 5, which was also used in our past research (Kim et al., Nature Biomedical Engineering, 4(11), 1053–1062). For the pharmacokinetics (PK) experiment in rabbits, previous research with Dutch Belted rabbits and a specific difference of 10 ng/g as the smallest difference that is important to detect has shown differences between brimonidine and brimonidine-vehicle-based formulations of approximately 42 ng/g drug concentration at 7 days with a standard deviation of 8.57 ng/g. Calculation using 5% type 1 error and 80% power supports a group size of n = 4. It should be noted that no group comparisons were made in this PK experiment.
Data exclusions	In the peptide microarray experiment, the PEPperPRINT protocol tolerated a maximum spot-to-spot deviation of 40%. Otherwise, the corresponding intensity value was reported as zeroed. No data were excluded in the analytical sample analysis. No data were excluded in the rabbit IOP studies. In the pharmacokinetic study, one data point was excluded from the day-1 iris tissue group due to an error in sample collection.
Replication	All protocols and methods have been successfully performed and repeated by 2 or more authors listed in the paper. After confirming the details, all experiment parameters and procedures were confirmed and written in the material and methods section.
Randomization	The rabbits used in this study were selected based on uniform sex distribution and were randomly assigned to each group. The IOP/safety studies were conducted with a gender composition of 3 males and 2 females, while the pharmacokinetic study was conducted with 2 males

	and 2 females per each time point. For the in vitro analytical experiments (in vitro melanin binding assay, cathepsin assay, cell uptake assay, stability assay, LC-MS samples), randomizing the order of sample analysis on the labeled samples would not impact the results of the measurements. When experiments were replicated or split up over multiple days, there were always replicates from each group and controls included.
Blinding	Masking procedures were implemented in studies as detailed in the Methods section. To ensure accuracy, a masked observer confirmed the IOP measurements for each rabbit at every time point, while the ophthalmologist conducting safety evaluations remained masked to the treatment. For the in vitro analytical experiments (in vitro melanin binding assay, cathepsin assay, cell uptake assay, stability assay), masking was not applied. Multiple investigators were involved at each step of the experimental protocols and sample analyses, reducing the likelihood of bias. The analytical methods used are inherently independent of user bias.

Reporting for specific materials, systems and methods

We require information from authors about some types of materials, experimental systems and methods used in many studies. Here, indicate whether each material, system or method listed is relevant to your study. If you are not sure if a list item applies to your research, read the appropriate section before selecting a response.

Materials & experimental systems

n/a	Involved in the study
☐	☒ Antibodies
☐	☒ Eukaryotic cell lines
☒	☐ Palaeontology and archaeology
☐	☒ Animals and other organisms
☒	☐ Clinical data
☒	☐ Dual use research of concern

Methods

n/a	Involved in the study
☒	☐ ChIP-seq
☒	☐ Flow cytometry
☒	☐ MRI-based neuroimaging

Antibodies

Antibodies used	Mouse monoclonal anti-HA (12CA5) DyLight800. Here is the detail information, manufacturer: BioxCell & PEPperPrint; catalogue numbers: #RT0268, PEPperCHIP® Mouse Monoclonal anti-HA (12CA5)-DyLight800 Control; 1:2000 dilution or 0.5 µg/ml
Validation	The antibody was used as a control for binding to the microarray. Pre-staning of microarray showed consistent binding to the HA controls (YPYDVPDYAG, 68 spots), which satisfied the PepPerPrint quality control criteria. To clarify, this antibody was not used to generate any data in this paper.

Eukaryotic cell lines

Policy information about cell lines and Sex and Gender in Research

Cell line source(s)	ARPE-19 (CRL-2302, lot No. 70013110) from American Type Culture Collection (ATCC).
Authentication	Cell lines were used as provided with authentication from the ATCC. See quality control specification from the following link: https://www.atcc.org/products/crl-2302?matchtype=&network=g&device=c&adposition=&keyword=&gclid=Cj0KCQjw8e-gBhD0ARIsAJiDsaX-uWjUwNjZ94xV5JNFOH4vVx5ZW4t1-pAGXep2nCnitZ5dBw9RYeAaAl7jEALw_wcB<br>STR profiling:<br>Amelogenin: X,Y<br>CSF1PO: 11<br>D13S317: 11,12<br>D16S539: 9,11<br>D5S818: 13<br>D7S820: 9,11<br>THO1: 6,9.3<br>TPOX: 9,11<br>vWA: 16,19
Mycoplasma contamination	The ARPE-19 cell line was directly purchased from ATCC. No mycoplasma contamination was reported.
Commonly misidentified lines<br>(See ICLAC register)	None.

Animals and other research organisms

Policy information about studies involving animals; ARRIVE guidelines recommended for reporting animal research, and Sex and Gender in Research

Laboratory animals	Dutch Belted Rabbits (4–5 months).

Wild animals	No wild animals were used in this study.
Reporting on sex	The randomization and inclusion of equal numbers of male and female animals is described in the methods.
Field-collected samples	No field-collected samples were used in this study.
Ethics oversight	All experimental protocols were approved by the Johns Hopkins Animal Care and Use Committee. All animals were handled and treated in accordance with the Association for Research in Vision and Ophthalmology Statement for Use of Animals in Ophthalmic and Vision Research.

Note that full information on the approval of the study protocol must also be provided in the manuscript.
